# The Diagnosis and Treatment of Fractures

**Published:** 1910-08

**Authors:** Frank K. Boland

**Affiliations:** Atlanta, Georgia; Professor of Operative and Clinical Surgery, Atlanta School of Medicine


### Journal-Record of Medicine

Successor to Atlanta Medical and Surgical Journal, Established 1855, and Southern Medical Record, Established 1870.

**OWNED BY THE ATLANTA MEDICAL JOURNAL CO.**

**Published Monthly**

*Official Organ Fulton County Medical Society, State Examining Board, Presbyterian Hospital, Atlanta, Birmingham and Atlantic Railroad Surgeons' Association, Chattahoochee Valley Medical and Surgical Association, Etc.*

EDGAR G. BALLENGER., M. D., Editor.
BERNARD WOLFF, M. D., Supervising Editor.
A. W. STIRLING, M. D., C. M., D. P. H., J. S. HURT, B. Ph., M. D.
GEO. M. NILES, M. D., W. J. LOVE, M. D., (Ala.); Associate Editors.
E. W. ALLEN, Business Manager.

COLLABORATORS

Dr. W. F. WESTMORLAND, General Surgery.
F. W. McRAE, M. D., Abdominal Surgery.
H. F. HARRIS, M. D., Pathology and Bacteriology.
E. B. BLOCK, M. D., Diseases of the Nervous System.
MICHAEL HOKE, M. D., Orthopedic Surgery.
CYRUS W. STRICKLER, M. D., Legal Medicine and Medical Legislation.
E. C. DAVIS, A. B., M. D., Obstetrics.
E. G. JONES, A. B., M. D., Gynecology.
R. T. DORSEY, Jr., B. S. M. D., Medicine.
L. M. GAINES, A. B., M. D., Internal Medicine.
J. N. LeCONTE, M. D., Disease of the Stomach and Intestines.
L. B. CLARKE, M. D., Pediatrics.
EDGAR PAULIN, M. D., Opsonic Medicine.
THEODORE TOEPEL, M. D., Mechano Therapy.
R. R. DALY, M. D., Medical Society.
A. W. STIRLING, M. D., etc., Diseases of the Eye, Ear, Nose and Throat.
BERNARD WOLFF, M. D., Diseases of the Skin.
E. G. BALLENGER, M. D., Diseases of the Genito-Urinary Organs.

VOL. LVI. AUGUST, 1910 No. 5.

INDEX THROUGH

### THE DIAGNOSIS AND TREATMENT OF FRACTURES.

FRANK K. BOLAND, M. D.,

*Professor of Operative and Clinical Surgery, Atlanta School of Medicine.*

While we have learned a great deal in recent years about the prophylaxis of many diseases, it is not probable that we will ever learn anything about the prophylaxis of fractures. Indeed, in this day of automobiles and aeroplanes, the injury must be on the increase. Already, it is estimated to make up one-seventh of all injuries.

Therefore, if we are unable to prevent the lesion, we should constantly strive to perfect ourselves in its diagnosis and treatment. I believe that success in this line depends more on the painstaking fulfilment of the rules already established than on the discovery of new ones. Surgeons are prone to look upon fractures as belonging to the domain of minor surgery, and turn them over to assistants and hospital internes. The proper management of a bad fracture requires as much skill and experience as the performance of many major surgical operations.

Conscientious work is imperative for satisfactory results in these cases, and while some fractures turn out badly in spite of the utmost care, there can be no doubt that the man who handles each case to the best of his ability, and sees it through until it is well, will, in the end, have a record in fractures of which he need not be ashamed.

When I first became a hospital interne, fresh from college, I don't think I had ever seen a recent fracture in my life. As this hospital admitted a great many accident cases, it frightened me to think of having sometimes to treat a fracture on my own responsibility. I had heard so much at school of the poor results obtained in fractures, and of the law-suits following them. Accordingly, I learned to approach every such injury with the profoundest respect, a feeling which I am glad to say I have not outgrown.

While I could not expect to bring before this body anything new on the subject of fractures, yet on account of the frequency of the lesion, and because it is not being given the attention which it demands, it will not be amiss to emphasize a few points which seem especially significant to me.

I question the propriety of two familiar terms used in the description of fractures. These are "simple" and "compound." While their meaning is understood by the practitioner, to the student meeting them for the first time, they are confusing and misleading. "Simple fracture" conveys the idea that there is simply a fracture and nothing more, whereas in a large proportion of cases we know that there is considerably more damage done than the break of the bone. It is hard to conceive of a blow powerful enough to sever a bone which will not at the same time badly bruise and tear muscles and fascia. A fracture due to indirect violence may be an exception, and of course if important vessels, nerves or viscera are injured the variety of fracture is modified by the term "complicated."

This is not my main objection to "simple fracture," however. The chief reason for considering it an inappropriate term is that it fails to express the opposite of "compound fracture," as it is supposed to do. To do this clearly the word "simple" should be changed to "closed," and for the same reason "compound" should be changed to "open." "Compound fracture" puzzles the student because very naturally he associates it with "complicated," etc.

A working knowledge of the anatomy which we learn in our Freshman year, and which we are apt to think is unduly stressed, often becomes extremely useful in handling a fracture. This is the anatomy of bones and muscles, the study of which goes hand in hand. Take, for example, that troublesome member, the supinator brevis. In a fracture of the upper third of the radius it is of prime consequence to recall how the insertion of this muscle hugs the neck of the radius, and maintains it in supination, while the pronators are holding the lower fragment in pronation. Since the upper fragment is so small and deeply situated that we cannot influence its position, while the position of the lower fragment can be regulated, the injury must be fixed with the forearm in extreme supination to accommodate the upper fragment.

In the same way, in a fracture of the upper third of the femur, it is important to remember the insertion of the powerful ilio-psoas muscle into the lesser trochanter. This muscle tilts the upper fragment forward, and as it is so situated that we cannot handle it, the lower fragment must be brought into line with it, to do which the thigh must be put upon an inclined plane.

In examining an injured extremity bear in mind that, as in other lesions, a fracture may exist in the absence of many of the so-called classical signs. Several things can prevent crepitation, i. e., overlapping, interposition of soft tissue, wide separation of the parts. An incomplete fracture will not give abnormal mobility. A point or line of exquisite tenderness is a valuable sign of this variety.

The X-ray does not possess the great value in the diagnosis and treatment of fractures usually accorded it, unless there is an expert to take the skiagram and read it. A large class of patients have come to require the X-ray in fractures because they believe it is the proper thing, whereas, often the physician is aided very little, if any, by it.

Anesthesia is an adjunct in the diagnosis and treatment of fractures of far more practical good to the average man than the X-ray. I have almost come to think that every fracture case should be anesthetized in order to assure the best results. On account of the pain and rigidity of muscles, it is impossible to get the most accurate diagnosis and most complete reduction without anesthesia. A Colles' fracture, one of the commonest we have, is an example of this. The reduction of this fracture is decidedly more important than the application of any kind of retentive splint. If it is thoroughly reduced, it is difficult for it again to become displaced except by extreme violence. Nitrous oxide gas is unavailable in the reduction of fractures since it does not produce relaxation.

It should be superfluous in this day to warn against the tight application of splints, and yet only recently I was compelled to do a shoulder-joint amputation for gangrene from this cause. I do not advocate the use of the circular plaster-of-Paris bandage in the first treatment of fractures. If plaster is preferred, it is best to use moulded longitudinal splints until the swelling has subsided.

I have lately seen fractures of the lower extremities treated without extension, and distressing results obtained thereby. We can't expect to avoid overlapping and shortening in these cases without the use of some kind of extension apparatus. And this leads me to say that if extension is not employed in a fracture of the shaft of the femur, we have a case calling for operative treatment. There are three bones in which fracture should be submitted to immediate open operation, provided the necessary aseptic surroundings are obtainable. These are the femur, humerus and patella. It seems impossible to get perfect results in these cases by any other means.

The ambulatory treatment of fractures is not usually practicable on account of the special apparatus which is necessary. The after-results have been unsatisfactory, since secondary shortening and excessive callus formation are frequent.

To sum up, the profession is not taking the proper interest in the diagnosis and treatment of fractures, nothing like it is in abdominal surgery, for instance. And certainly, there is no department of surgery more important than fractures. The whole key to success in the matter is careful, thorough work in each individual case.

